# Supplementary material for: The double-stranded DNA-binding proteins TEBP-1 and TEBP-2 form a telomeric complex with POT-1
Source: Nat Commun. 2021 May 11;12:2668. doi: 10.1038/s41467-021-22861-2 (PMC8113555; doi:10.1038/s41467-021-22861-2)
Supplement: Supplementary file 1 — Supplementary Information [file 41467_2021_22861_MOESM1_ESM.pdf]

SUPPLEMENTARY INFORMATION

The double-stranded DNA-binding proteins TEBP-1 and TEBP-2 form a telomeric complex with POT-1

Sabrina Dietz[1,5], Miguel Vasconcelos Almeida[1,2,5], Emily Nischwitz[1], Jan Schreier[1], Nikenza Viceconte[1], Albert Fradera-Sola[1], Christian Renz[1], Alejandro Ceron-Noriega[1], Helle D. Ulrich[1], Dennis Kappei[3,4], René F. Ketting[1], Falk Butter[1,*]

[1]Institute of Molecular Biology (IMB), Ackermannweg 4, 55128 Mainz, Germany

[2]Present address: Wellcome Trust/Cancer Research UK Gurdon Institute, University of Cambridge, Tennis Court Road, Cambridge, CB2 1QN, UK; and Department of Genetics, University of Cambridge, Downing Street, Cambridge CB2 3EH, UK.

[3]Cancer Science Institute of Singapore, National University of Singapore, 117599, Singapore

[4]Department of Biochemistry, Yong Loo Lin School of Medicine, National University of Singapore, 117596 Singapore

[5]These two authors contributed equally to this work

*Correspondence: f.butter@imb-mainz.de, +49 6131-39-21570

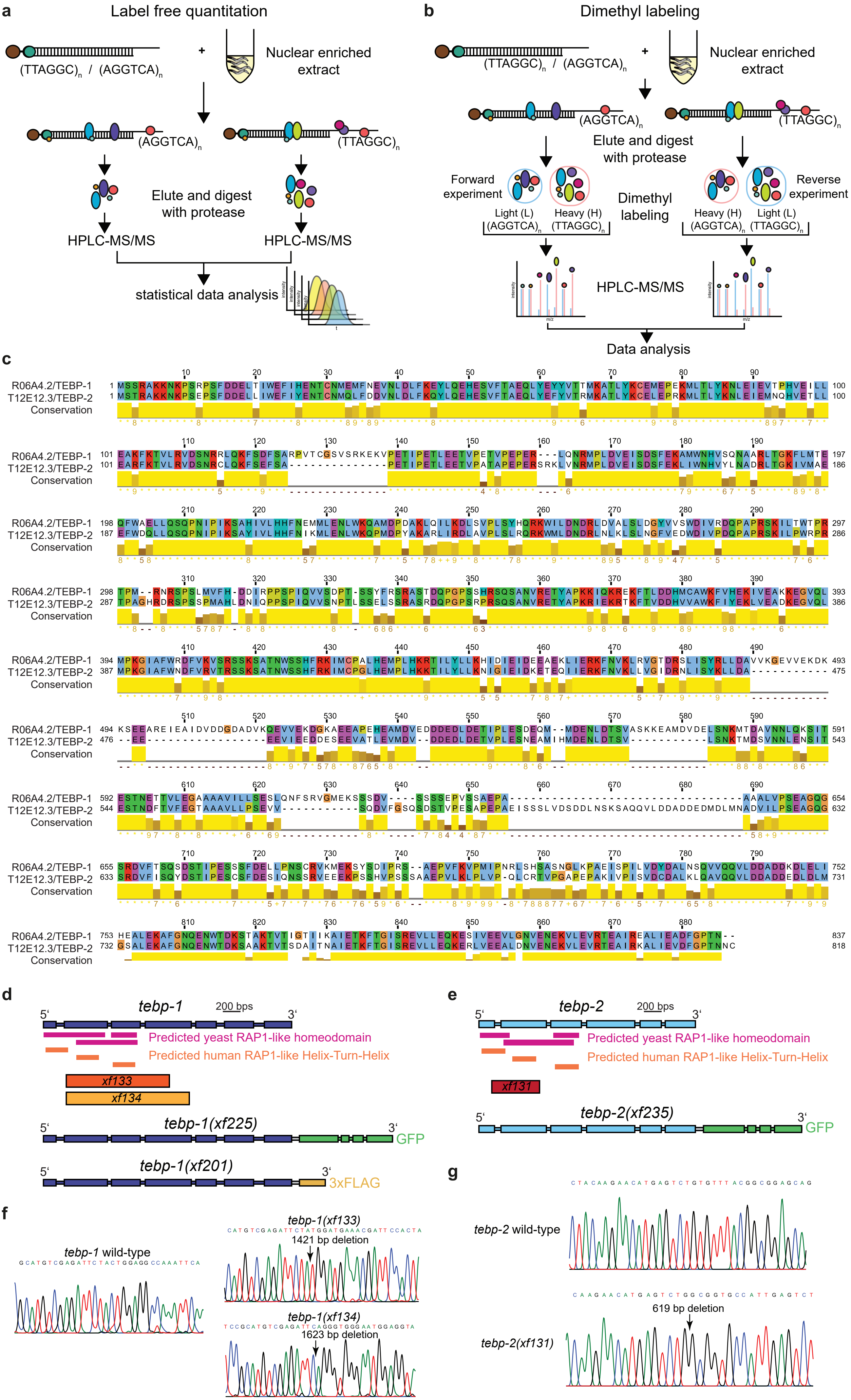

Supplementary Fig. 1

Supplementary Fig. 1. A quantitative proteomics screen for telomere binders identifies the paralogs TEBP-1 and TEBP-2.

(a) Scheme representing the label free quantitation workflow. Telomere $(TTAGGC)_n$, or control DNA $(AGGTCA)_n$ baits are incubated with nuclear extract. Samples are processed and measured independently, and later compared by statistical data analysis.

(b) Scheme representing the reductive dimethyl labeling workflow. Telomere $(TTAGGC)_n$, or control DNA $(AGGTCA)_n$ baits are incubated with nuclear extract in duplicates. Per condition each peptide gets labeled with either light methyl groups (CH_3) or heavy methyl groups (CD_3). Afterwards, the heavy sample of one condition is combined with the light sample of the other condition and vice-versa to achieve a forward and a reverse experiment. Forward and reverse experiments are measured and analyzed by comparing intensities of the proteins (calculated from their peptide intensities) in the respective channel.

(c) Pairwise sequence alignment of amino acid sequences of TEBP-1 and TEBP-2 using EMBOSS Needle, visualized using Jalview, showing the high sequence similarity between the two proteins. Amino acids are color coded according to the Clustal X colour scheme: blue – amino acids A, I, L, M, F, W, C and V; red: amino acids R and K; green – amino acids N, S, Q, T; pink – amino acid C; magenta – amino acids E and D; orange – amino acid G; cyan – amino acids H, Y; yellow – amino acid P. Conservation is shown in the yellow bars beneath the sequences, brighter yellow for higher conservation. Amino acid positions are indicated.

(d) Scheme of the *tebp-1* genomic locus. Below are indicated the positions with similarity to the homeodomain of human and yeast RAP1, as predicted by HHPred (3.2.0), deletions made by CRISPR-Cas9 genome editing (alleles *xf133* and *xf134*), as well as the locations of the tags (C-terminal GFP and 3xFLAG), also inserted by CRISPRCas9 genome editing.

(e) As in (d) but for the *tebp-2* locus.

(f-g) Chromatograms of Sanger sequencing of *tebp-1* and *tebp-2* deletion alleles compared to WT. Deletion sites are indicated with arrows. Colors indicate the different DNA bases: black – G; blue – C; red – T; green – A.

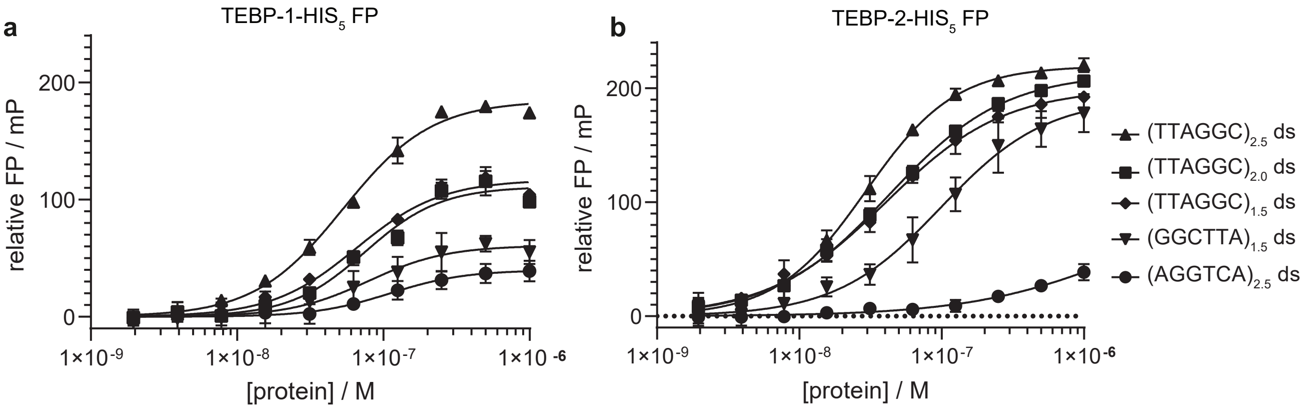

c

Protein	Oligo Sequence	K_d	B_{max}
TEBP-1-HIS_5	$(TTAGGC)_{2.5}$ ds	53.05 nM	184.5
TEBP-1-HIS_5	$(TTAGGC)_{2.0}$ ds	75.25 nM	111.1
TEBP-1-HIS_5	$(TTAGGC)_{1.5}$ ds	65.26 nM	117.0
TEBP-1-HIS_5	$(GGCTTA)_{1.5}$ ds	78.74 nM	60.80
TEBP-1-HIS_5	$(AGGTCA)_{2.5}$ ds	110.6 nM	39.88
TEBP-2-HIS_5	$(TTAGGC)_{2.5}$ ds	29.37 nM	219.6
TEBP-2-HIS_5	$(TTAGGC)_{2.0}$ ds	42.77 nM	213.2
TEBP-2-HIS_5	$(TTAGGC)_{1.5}$ ds	40.53 nM	199.8
TEBP-2-HIS_5	$(GGCTTA)_{1.5}$ ds	99.08 nM	191.3
TEBP-2-HIS_5	$(AGGTCA)_{2.5}$ ds	195.4 nM	104.9

Supplementary Fig. 2

Supplementary Fig. 2. Telomeric double-strand binding preferences of TEBP-1 (R06A4.2) and TEBP-2 (T12E12.3).

(a-b) Fluorescence polarization assays of 1 µM to 2 nM purified TEBP-1-His_5 (a) and TEBP-2-His_5 (b). Proteins were incubated with 2.5x, 2.0x, 1.5x T-rich, and 1.5x G-rich double-stranded telomeric FITC-labeled oligonucleotides, as well as 2.5x double-stranded control. Error bars represent +/- the standard deviation of the mean values. Per data point n=3 technical replicates. FP, fluorescence polarization; mP, millipolarization, upward triangle: 2.5x TTAGGC double-strand, downward triangle: 2.5x TTAGGC single-strand, square: 2x TTAGGC double-strand, diamond: 1.5x TTAGGC T-rich double-strand, downward triangle: 1.5x G-rich GGCTAA double-strand, circle: 2.5x shuffled control AGGTCA double-strand.

(c) Overview of K_d and B_{max} values from FP experiment (a-b).

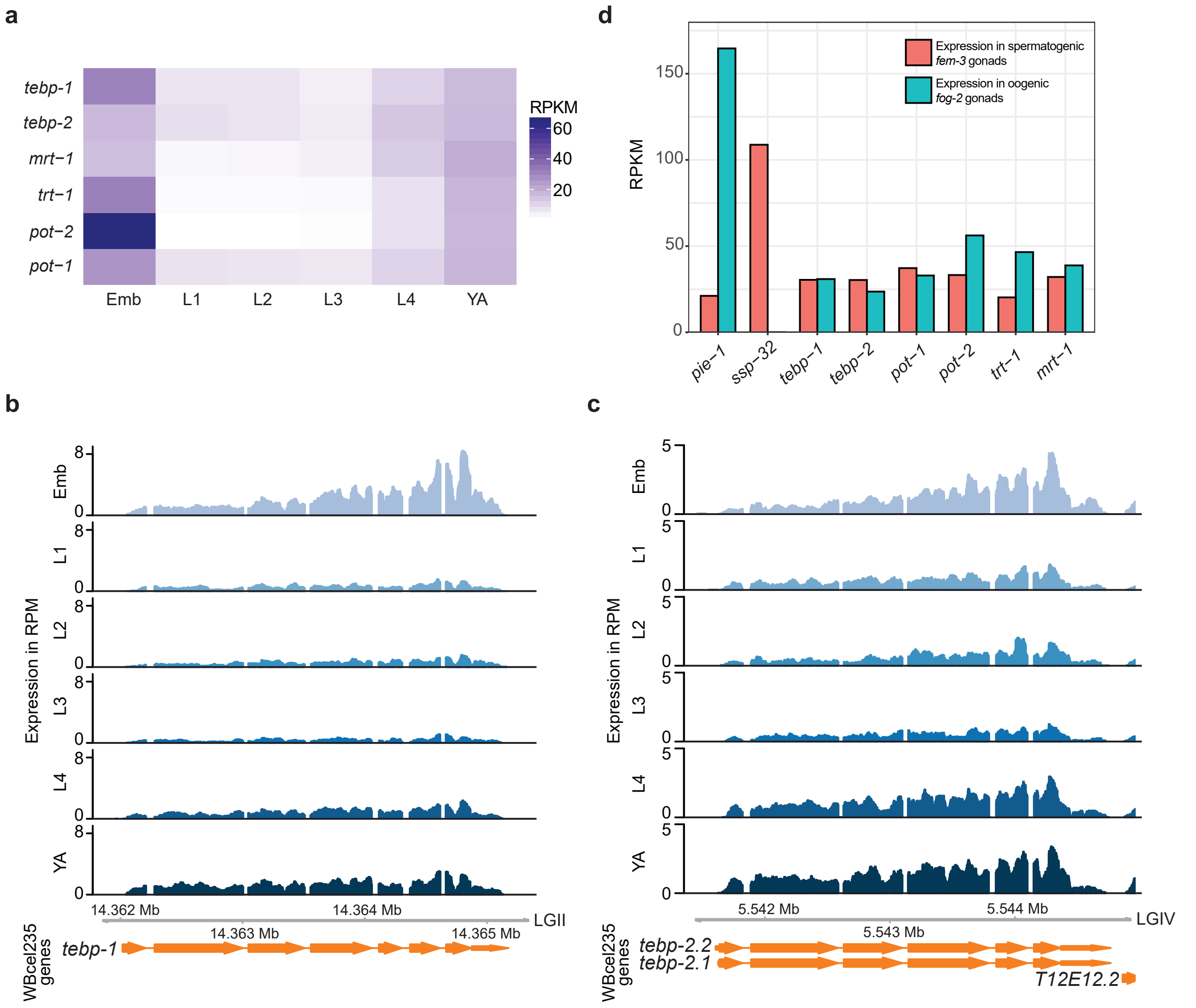

Supplementary Fig. 3

Supplementary Fig. 3. The expression profiles of *tebp-1* and *tebp-2* throughout development and in isolated gonads.

(a) Heatmap depicting mRNA expression levels, in Reads Per Kilobase Million (RPKM), of the known telomere binders *pot-1*, *pot-2*, and *mrt-1*, telomerase subunit *trt-1*, as well as *tebp-1* and *tebp-2*. Data from a previously published RNA-seq dataset[47].

(b-c) Genome browser tracks with the mRNA expression of *tebp-1* (b), and *tebp-2* (c), in reads per million (RPM), across the different life stages of *C. elegans*. Data from [47]. (a-c) Emb, embryos; L1-L4, first to fourth larval stages; YA, young adults.

(d) Expression of telomere factors in dissected *fem-3* mutant gonads (exclusively spermatogenic) and *fog-2* mutant gonads (exclusively oogenic), from previously published RNA-seq data[48]. *pie-1* and *ssp-32* are genes known to be expressed in oogenesis and in spermatogenesis, respectively, according to [48].

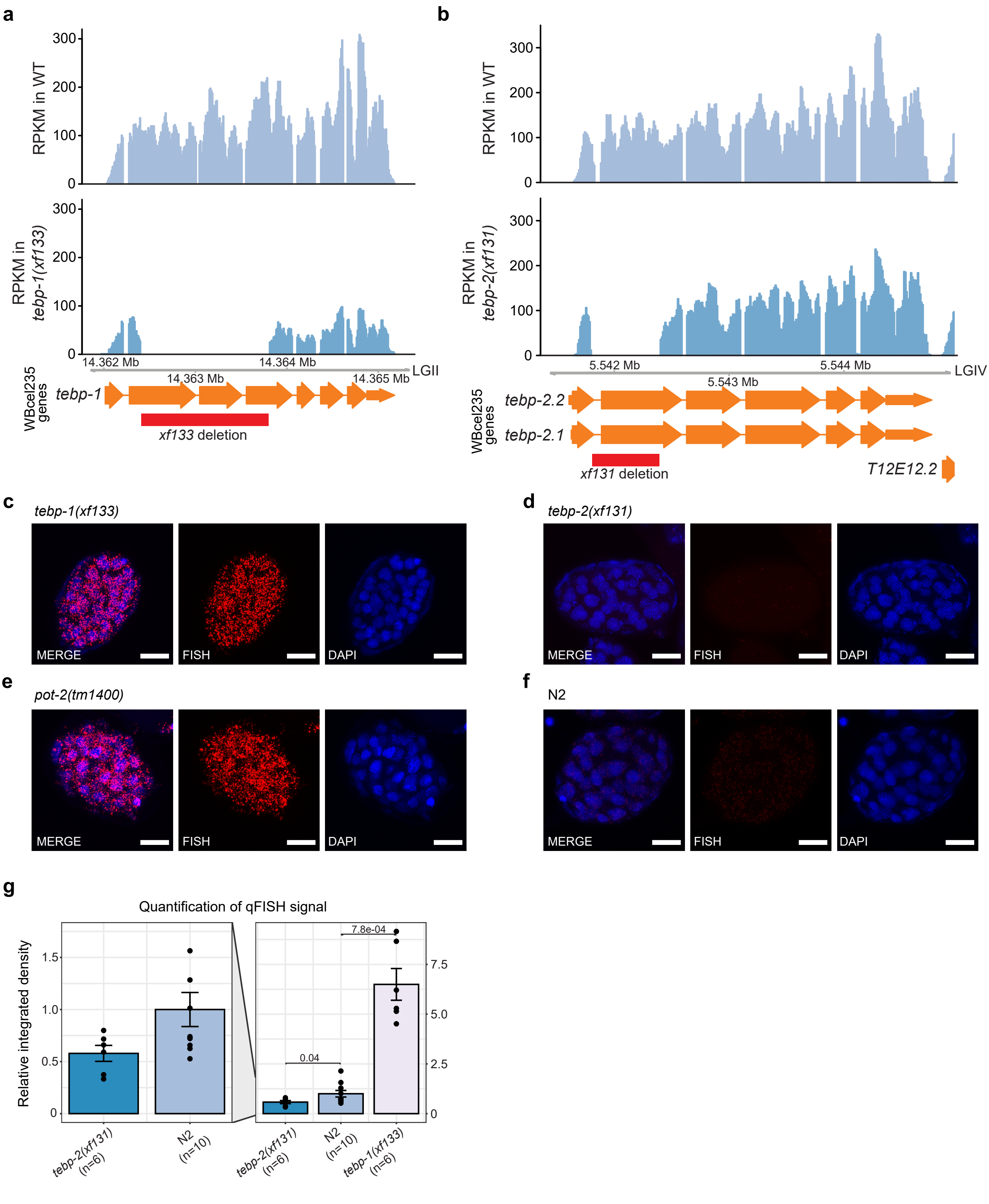

Supplementary Fig. 4

Supplementary Fig. 4. TEBP-1 and TEBP-2 regulate telomere length in embryos.

(a-b) Genome browser tracks with the mRNA expression of *tebp-1* (a) and *tebp-2* (b), in Reads Per Kilobase Million (RPKM). RNA-seq data of wild-type, *tebp-1(xf133)*, and *tebp-2(xf131)* mutants.

(c-f) Representative maximum projection z-stacks of a qFISH assay using embryos of *C. elegans* mutant strains. The telomeres of these embryos were visualized by hybridization with a telomeric PNA-FISH-probe. Nuclei were stained with DAPI. Scale bars, 10 µm. *tebp-1(xf133)* and *tebp-2(xf131)* were grown for approx. 98/120 generations before the experiment. N = 3 biologically independent experiments with similar results.

(g) Barplot depicting analysis of qFISH images of the strains in (c-f), as indicated on the x-axis. Average telomere length is indicated by arbitrary units of relative integrated density on the y-axis, with wild-type N2 set to 1. The left hand plot is a zoomed-in inset of the N2 and *tebp-2(xf131)* values. n of analyzed independent embryos per strain: *tebp-2(xf131)*: n=6, N2: n=10, *tebp-1(xf131)*: n=6. Error bars represent the standard error of the mean (SEM) and p-values were calculated using Welch’s t-test.

a

Genotype	Father	Mother	Synthetic sterile F2	Could grow double mutant homozygous line
tebp-2(xf131); tebp-1(xf133)	*tebp-2(xf131)*	*tebp-1(xf133)*	Yes	No
tebp-1(xf133); tebp-2(xf131); xfIs148(tebp-2::gfp MosSCI)	*tebp-1(xf133)*	*tebp-2(xf131); xfIs148(tebp-2::gfp MosSCI)*	No	Yes
tebp-2(xf131); tebp-1(xf133)	*tebp-1(xf133)*	*tebp-2(xf131); xfIs148(tebp-2::gfp MosSCI)*	Yes	No
tebp-2(xf131); pot-2(tm1400)	*tebp-2(xf131)*	*pot-2(tm1400)*	No	Yes
tebp-1(xf133); trt-1(ok410)	*tebp-1(xf133)*	*trt-1(ok410)*	No	Yes
tebp-1(xf133); mrt-1(tm1354)	*tebp-1(xf133)*	*mrt-1(tm1354)*	No	Yes
pot-2(tm1400); trt-1(ok410)	*pot-2(tm1400)*	*trt-1(ok410)*	No	Yes
tebp-2(xf131); trt-1(ok410)	*tebp-2(xf131)*	*trt-1(ok410)*	No	Yes
tebp-1(xf260); pot-2(tm1400)	N/A. Used CRISPR-Cas9 to introduce *tebp-1* mutation due to linkage	*pot-2(tm1400)*	N/A not a cross	Yes

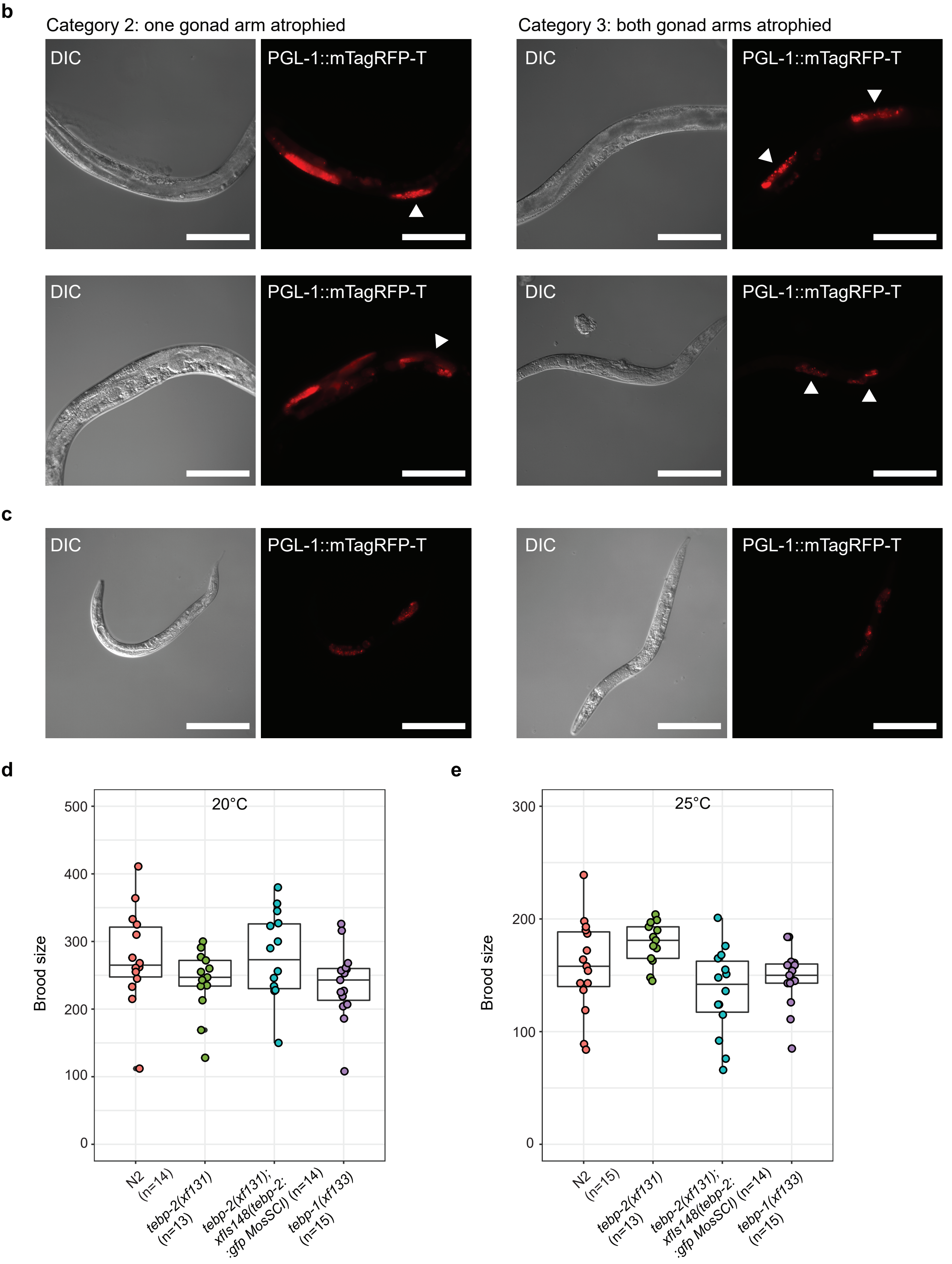

Supplementary Fig. 5

Supplementary Fig. 5. Dissecting the role of TEBP-1 and TEBP-2 in fertility.

(a) Overview of additional crosses performed to investigate distinct aspects of the synthetic sterility phenotype. For each cross, the columns indicate the genotype of the animals analyzed, the genotype of their parents, whether the animals have synthetic sterility, and if we could establish a homozygous line. The second row shows that the reciprocal cross between *tebp-1* and *tebp-2* also led to synthetic sterility. The third row shows that a *tebp-2::gfp* single-copy transgene rescues the synthetic sterility of *tebp-1; tebp-2* double mutants, while their transgene-less siblings still display synthetic sterility (fourth row). The following rows demonstrate that the synthetic sterility is specific to *tebp-1* and *tebp-2*, as it does not arise in crosses with other telomere-associated mutants.

(b) Additional representative widefield DIC and fluorescence pictures of worms with germlines of categories 2 (left panels) and 3 (right panels). Scale bars, 200 µm. Atrophied germlines are indicated with white arrowheads.

(c) Exemplary widefield DIC and fluorescence micrographs of worms showing growth defects and/or larval arrest. These animals were isolated concurrently to animals shown in (b), but did not reach adulthood. These two specific animals were offspring of *tebp-2(xf131); tebp-1(xf133)* +/-. Scale bars, 200 µm.

(d-e) Boxplots showing the brood sizes of wild-type N2, *tebp-1* or *tebp-2* single mutants, and *tebp-2(xf131); xfIs148(tebp-2::gfp)*. Central horizontal lines represent the median, the bottom and top of the box represent the 25th and 75th percentile, respectively. Whiskers represent the 5th and 95th percentile, dots represent the data points used to calculate the box plot. Experiments were carried out at 20°C (d) and 25°C (e). Statistical comparisons were performed with wildtype N2, calculated with two-sided and unpaired Mann–Whitney and Wilcoxon tests. N2 vs.*tebp-2(xf131)*: 20°C p-value=0.145, 25°C p-value= 0.097; N2 vs. *tebp-2(xf131);xfIs148(tebp-2::gfp MosSCI)*: 20°C p-value=0.91, 25°C p-value=0.183; N2 vs. *tebp-1(xf133)*: 20°C p-value=0.052, 25°C p-value=0.41. Analyzed individuals per strain are indicated as n on the x-axis labels.

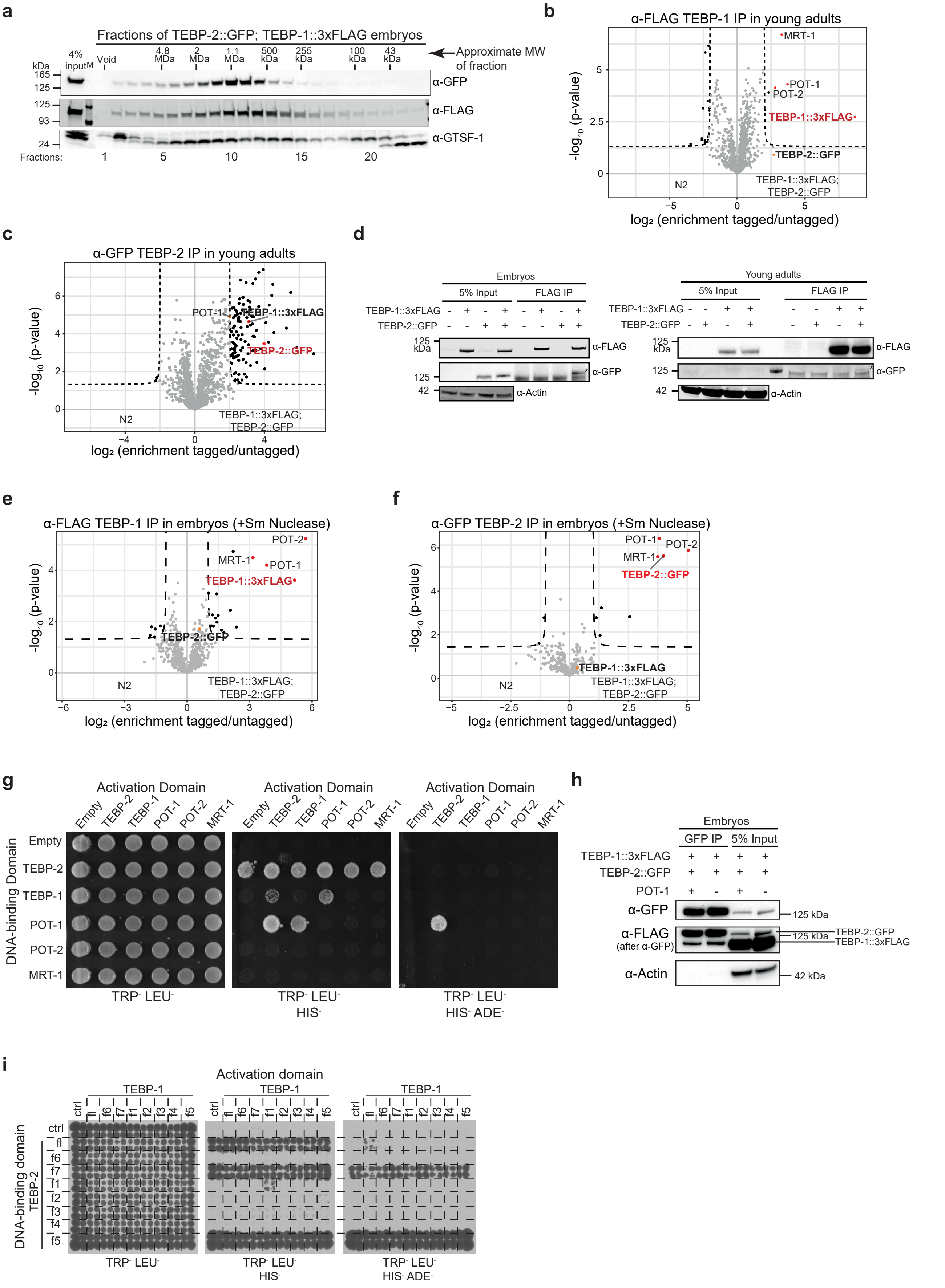

Supplementary Fig. 6

Supplementary Fig. 6. TEBP-1 and TEBP-2 interact with each other and with POT-1/MRT-1/POT-2.

(a) Western blot of the eluted fractions from size-exclusion chromatography of embryo extracts containing TEBP-1::3xFLAG and TEBP-2::GFP. The approximate molecular weight (MW) of the fractions is indicated above the blots. GTSF-1 was used as a control, as it has a known elution profile in size-exclusion chromatography[76]. Information about α-GTSF-1 can be found in [76]. N = 2 biologically independent experiments with similar results.

(b-c) Volcano plots with quantitative proteomic analysis of TEBP-1::3xFLAG (b) or TEBP-2::GFP (c) IPs in young adults. IPs were performed in quadruplicates. Enriched proteins (threshold: 4-fold, p-value<0.05) are shown as black dots, enriched proteins of interest are highlighted with red or orange dots, and the baits are named in red. Background proteins are depicted as grey dots.

(d) Co-IP western blot experiment of TEBP-1::3xFLAG and TEBP-2::GFP similar to Fig. 5e-f, except the IPs were performed with an α-FLAG antibody. Actin was used as loading control. IPs with embryo extracts in the left panel and with young adult extracts in the right panel. N = 3 biologically independent experiments with similar results for both experiments.

(e-f) Volcano plots showing quantitative proteomic analysis of either TEBP-1::3xFLAG (e) or TEBP-2::GFP (f) IPs in embryos. IPs were performed in quadruplicates and Sm nuclease was added to remove potential DNA-dependent interactions. Enriched proteins (threshold >2-fold, p-value<0.05) are shown as black dots. Enriched proteins of interest are highlighted with red or orange dots, and baits are named in red.

(g) Orthogonal grid of the Y2H spotting containing fusion constructs of the Gal4 activating or DNA-binding domains with the full length sequence of telomere factors. Left panel shows growth control in non-restrictive medium. Protein-protein interactions allow for growth on TRP- LEU- HIS- medium (middle panel). TEBP-2 bound to the Gal4 DNA-binding domain is self-activating, precluding the determination of interactions. The strongest interactions are permissive of growth on the highly stringent TRP- LEU- HIS- ADE- medium (right panel).

(h) Co-IP western blot experiments of TEBP-1::3xFLAG and TEBP-2::GFP in the presence and absence of POT-1, where absence of POT-1 refers to the *pot-1(tm1620)* mutation. The IPs were performed with an α-GFP antibody. Actin was used as loading control. IPs were performed with 800 µg of embryo extracts. Detection by ECL was performed sequentially, first for GFP and then for FLAG.

(i) Y2H spotting as in (g) with TEBP-1 and TEBP-2 partial constructs fused to the GAL4 activation or DNA-binding domain as in Fig. 6d,h. The full length, f7, and f5 TEBP-2 constructs fused to the Gal4 DNA-binding domain show self-activation. As in (g) the growth on the highly stringent TRP-LEU-HIS-ADE-medium (right panel) indicates the strongest interactions.

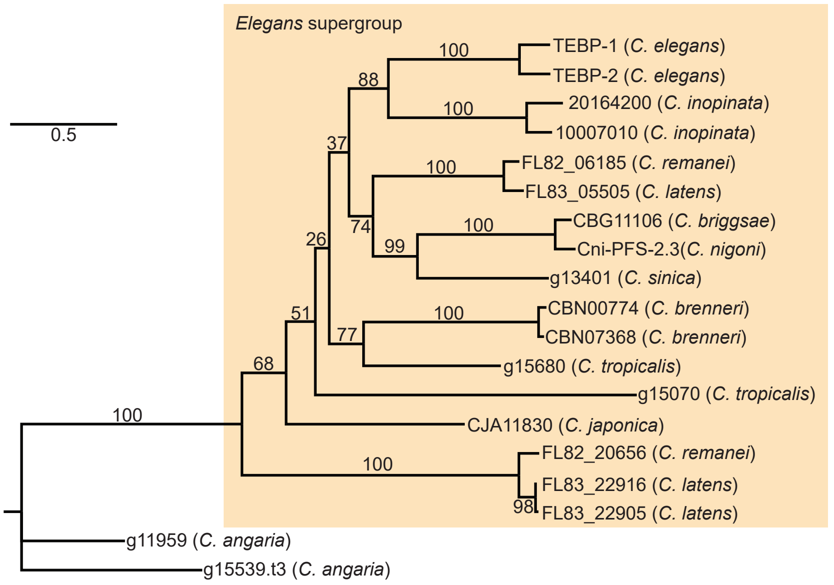

Supplementary Fig. 7

Supplementary Fig. 7. Phylogenetic analysis of the N-terminal region of TEBP-proteins.

Phylogenetic tree constructed as in Fig. 7a. The MAFFT protein alignment used for this tree comprised the first 600 alignment positions of the multiple sequence alignment in Supplementary Data 2 (sheet 2). Values on the nodes represent bootstrapping values of 10000 replicates, set to 100.

Supplementary Table 1: List of strains used and created in this study.

Listed are all strains with their respective genotype and source.

Strain Reference	Genotype	Source
	Wild-Type N2	CGC
YA1197	*ypIn2 [daz-1p::pot-1::mCherry::tbb-2 3'UTR + Cbr-unc-119(+)] II.*	A kind gift from Shawn Ahmed
tm1620	*pot-1(tm1620) III.*	National Bioresource Project for the nematode, Japan
tm1400	*pot-2(tm1400) II.*	National Bioresource Project for the nematode, Japan
YA1116	*mrt-1(tm1354) I.*	CGC
YA1059	*trt-1(ok410) I.*	CGC
EG6699	*ttTi5605 II; unc-119(ed3) III; oxEx1578*	CGC
RFK641	*tebp-2(xf131) IV.*	This study
RFK671	*tebp-1(xf133) II.*	This study
RFK672	*tebp-1(xf134) II.*	This study
RFK659	*TEBP-2(xfIs148[tebp-2(prm)::tebp-2::GFP::tebp-2(3'UTR)]) II; unc-119(ed9) III.*	This study
RFK1096	*tebp-2(xf235[TEBP-2::GFP]) IV.*	This study
RFK1022	*tebp-1(xf225[tebp-1::GFP]) II.*	This study
RFK958	*tebp-1(xf201[tebp-1::3xFLAG]) II.*	This study
RFK1173	*tebp-2(xf235[tebp-2::GFP]) IV; tebp-1(xf201[tebp-1::3xFLAG]) II.*	This study
RFK1174	*tebp-2(xf235[tebp-2::GFP]) IV; ypIn2[daz-1p::pot-1::mCherry::tbb-2 3'UTR + Cbr-unc-119(+)] II.*	This study
RFK1067	*tebp-1(xf225[tebp-1::GFP]) II; ypIn2[daz-1p::pot-1::mCherry::tbb-2 3'UTR + Cbr-unc-119(+)] II.*	This study
RFK1086	*pgl-1(xf233[pgl-1::mTagRFP-T]) IV.*	Jan Schreier, Ketting laboratory
RFK1327	*tebp-2(xf131) IV; pgl-1(xf233[pgl-1::mTagRFP-T]) IV.*	This study
RFK1328	*tebp-1(xf133) II; pgl-1(xf233[pgl-1::mTagRFP-T]) IV.*	This study
-	*tebp-2(xf131) IV; pot-2(tm1400) II.*	This study
-	*tebp-1(xf133) II; mrt-1(tm1354) I.*	This study
RFK1334	*trt-1(ok410) I; tebp-1(xf133) II.*	This study
RFK1309	*tebp-1(xf260) II; pot-2(tm1400) II.*	This study
-	*trt-1(ok410) I; pot-2(tm1400) II.*	This study
AF16	*C. briggsae* Wild-type	CGC

Supplementary Table 2. Fractions of the gel filtration runs and correlated molecular weight.

Separation range of the used column in red, fractions covered by the marker run in green. Fractions of the 96-well column marked in bold were concentrated and used for western blot detection (Figs. 5a and S6a respectively). MW: molecular weight.

Fraction	volume [ml]	log MW	calculated MW [kDa]	96 well
A1	1,0	8,982	960063,591	a1
A2	2,0	8,727	533212,105	a2
A3	3,0	8,472	296141,997	a3
A4	4,0	8,216	164475,040	a4
A5	5,0	7,961	91348,201	a5
A6	6,0	7,705	50734,105	a6
A7	6,5	7,578	37809,419	a7
A8	7,0	7,450	28177,340	**a8**
A9	7,5	7,322	20999,067	a9
A10	8,0	7,195	15649,483	**a10**
A11	8,5	7,067	11662,724	a11
A12	9,0	6,939	8691,605	**a12**
A13	9,5	6,811	6477,389	**b12**
A14	10,0	6,684	4827,252	**b11**
A15	10,5	6,556	3597,493	**b10**
A16	11,0	6,428	2681,020	**b9**
A17	11,5	6,301	1998,021	**b8**
A18	12,0	6,173	1489,018	**b7**
A19	12,5	6,045	1109,686	**b6**
A20	13,0	5,918	826,990	**b5**
A21	13,5	5,790	616,311	**b4**
A22	14,0	5,662	459,304	**b3**
A23	14,5	5,534	342,295	**b2**
A24	15,0	5,407	255,094	**b1**
A25	15,5	5,279	190,108	**c1**
A26	16,0	5,151	141,677	**c2**
A27	16,5	5,024	105,584	**c3**
A28	17,0	4,896	78,686	**c4**
A29	17,5	4,768	58,641	**c5**
A30	18,0	4,641	43,702	**c6**
A31	18,5	4,513	32,569	**c7**
A32	19,0	4,385	24,272	**c8**
A33	19,5	4,257	18,088	c9
A34	20,0	4,130	13,480	c10
A35	20,5	4,002	10,046	c11
A36	21,0	3,874	7,487	c12
A37	21,5	3,747	5,580	d12
A38	22,0	3,619	4,158	d11
A39	22,5	3,491	3,099	d10
A40	23,0	3,364	2,309	d9
A41	23,5	3,236	1,721	d8
A42	24,0	3,108	1,283	d7

Superose 6 column separation range (5-5000 kDa)

covered by marker run
