# Supplementary material for: The double-stranded DNA-binding proteins TEBP-1 and TEBP-2 form a telomeric complex with POT-1
Source: Nat Commun. 2021 May 11;12:2668. doi: 10.1038/s41467-021-22861-2 (PMC8113555; doi:10.1038/s41467-021-22861-2)
Supplement: Supplementary file 3 — Description of Additional Supplementary Files [file 41467_2021_22861_MOESM3_ESM.pdf]

Description of Additional Supplementary Files

File Name: Supplementary Data 1
Description: Results of HHPred (v. 3.2.0) runs with full length protein sequences of TEBP-1 and TEBP-2 showing the structural similarity to human and yeast RAP1 proteins.

File Name: Supplementary Data 2
Description: Evolutionary analysis of *tebp* orthologs in other *Caenorhabditis* species.

File Name: Supplementary Data 3
Description: Enrichment values for novel and previously described telomeric binders in *C. elegans* from the mass spectrometry screens in Figs. 1a,b and S1a,b. P-values for Label-free quantitation were calculated using Welch's t-test.

File Name: Supplementary Data 4
Description: Primers used in this study.

File Name: Supplementary Data 5
Description: Additional information for CRISPR-Cas9 genome editing, including protospacers, donor templates, injection mixes and plasmid vectors used in the creation of the tagged alleles and the *tebp-1;pot-2* double mutant.
