# Supplementary material for: The double-stranded DNA-binding proteins TEBP-1 and TEBP-2 form a telomeric complex with POT-1
Source: Nat Commun. 2021 May 11;12:2668. doi: 10.1038/s41467-021-22861-2 (PMC8113555; doi:10.1038/s41467-021-22861-2)
Supplement: Supplementary file 9 — Reporting Summary [file 41467_2021_22861_MOESM9_ESM.pdf]

nature research

Corresponding author(s): Falk Butter

Last updated by author(s): Mar 15, 2021

Reporting Summary

Nature Research wishes to improve the reproducibility of the work that we publish. This form provides structure for consistency and transparency in reporting. For further information on Nature Research policies, see our Editorial Policies and the Editorial Policy Checklist.

Statistics

For all statistical analyses, confirm that the following items are present in the figure legend, table legend, main text, or Methods section.

n/a	Confirmed	
☐	☒	The exact sample size (*n*) for each experimental group/condition, given as a discrete number and unit of measurement
☐	☒	A statement on whether measurements were taken from distinct samples or whether the same sample was measured repeatedly
☐	☒	The statistical test(s) used AND whether they are one- or two-sided<br>*Only common tests should be described solely by name; describe more complex techniques in the Methods section.*
☐	☒	A description of all covariates tested
☐	☒	A description of any assumptions or corrections, such as tests of normality and adjustment for multiple comparisons
☐	☒	A full description of the statistical parameters including central tendency (e.g. means) or other basic estimates (e.g. regression coefficient) AND variation (e.g. standard deviation) or associated estimates of uncertainty (e.g. confidence intervals)
☐	☒	For null hypothesis testing, the test statistic (e.g. *F*, *t*, *r*) with confidence intervals, effect sizes, degrees of freedom and *P* value noted<br>*Give P values as exact values whenever suitable.*
☒	☐	For Bayesian analysis, information on the choice of priors and Markov chain Monte Carlo settings
☐	☒	For hierarchical and complex designs, identification of the appropriate level for tests and full reporting of outcomes
☒	☐	Estimates of effect sizes (e.g. Cohen's *d*, Pearson's *r*), indicating how they were calculated

Our web collection on statistics for biologists contains articles on many of the points above.

Software and code

Policy information about availability of computer code

Data collection	MaxQuant quantitative proteomics software (version 1.5.2.8) - Cox & Mann, 2008<br>EMBOSS Needle (v. 6.6.0), Jalview (v. 2.11.0), HHPred 3.2.0, Fiji/ImageJ 1.53c, LasX microscopy software (Leica), Image Studio 3.1 (LI-COR), ImageLab V (BioRad), FastQC (v. 0.11.8), STAR aligner (v. 2.6.1b), featureCounts (v. 1.6.2), deepTools (v. 2.27.1), MAFFT (v. 7.452), IQ-Tree (v. 1.6.12), FigTree (v. 1.4.4)
Data analysis	Data analysis is described in detail in the Method section.<br>Analysis of data for MS, qFISH, germline scoring and fertility assays was performed with R (v.3.5.2/v 4.0.3) and included base packages as well as the following publicly available packages:<br>Volcano plots/Scatter plot MS: ggplot2 – v 3.2.1, ggrepel – v 0.8.1, stats – v 3.5.2<br>qFISH barplots: RColorBrewer - v 1.1-2, ggpubr - v 0.4.0, plyr - v 1.8.6, viridis - v 0.5.1, viridisLite - v 0.3.0, ggforce - v 0.3.2, ggsignif - v 0.6.0, dplyr - v 1.0.2, ggplot2 - v 3.3.3, readr - v 1.4.0<br>Brood size boxplots and germline category barplots: ggplot2 - v 3.2.1, reshape – v 0.8.8, viridis – v 0.5.1, scales – v 1.0.0<br>Vizualization of RNAseq data was done with Gviz (v.1.30.3) and GenomicFeatures (v.1.38.2)

For manuscripts utilizing custom algorithms or software that are central to the research but not yet described in published literature, software must be made available to editors and reviewers. We strongly encourage code deposition in a community repository (e.g. GitHub). See the Nature Research guidelines for submitting code & software for further information.

Data

Policy information about availability of data

All manuscripts must include a data availability statement. This statement should provide the following information, where applicable:

- Accession codes, unique identifiers, or web links for publicly available datasets
- A list of figures that have associated raw data
- A description of any restrictions on data availability

The datasets supporting the conclusions of this article are available in the ProteomeXchange Consortium via Pride repository, PXD019241 in http://www.ebi.ac.uk/pride/archive/projects/PXD019241; and in the SRA, BioProject PRJNA630690 in https://www.ncbi.nlm.nih.gov/bioproject/PRJNA630690.

Field-specific reporting

Please select the one below that is the best fit for your research. If you are not sure, read the appropriate sections before making your selection.

☒ Life sciences ☐ Behavioural & social sciences ☐ Ecological, evolutionary & environmental sciences

For a reference copy of the document with all sections, see nature.com/documents/nr-reporting-summary-flat.pdf

Life sciences study design

All studies must disclose on these points even when the disclosure is negative.

Sample size	Sample sizes for mass spectrometry experiments were established as quadruplicates (LFQ) or duplicates (DML), as is well-accepted in the proteomics field.<br>- results in Fig. 1a/b were derived from technical replicates<br>- results for all other IP-qMS experiments were derived from biological replicates (4 separate extract preparations per condition)<br><br>Sample sizes for the worm experiments were not based on statistical methods but on previously published similar experiments yielding consistent and reproducible results.<br>- IP experiments followed by Western blot: Embryo samples were prepared from bleaching one high density plate of gravid adults per sample, young adult samples were prepared from a pool of grown young adult worms aliquoted to 100 µl per sample.<br>- Colocalization was checked in separate animals (gravid adults, embryos) on different days and each showed similar results. Representative images from two life stages per strain are shown in the manuscript. A total of n>10 individuals was imaged for both strains.<br>- Telomere Southern Blot: per sample and strain gDNA derived from 100 µl mixed stage worms was used<br>- for qFISH sample size was not predetermined, images were taken to be around n=10-15 individual germlines/embryos per strain and selected before analysis according to pre-established criteria.<br>- for double mutant cross analysis >100 F2 were singled before genotyping and subsequent analysis (Fig. 4)<br>- brood sizes were determined from n=15 worms for Supplementary Fig 5 d/e, lower n resulting from excluding worms due to pre-established criteria<br>- brood sizes were determined blindly from n>50 worms for Fig. 4 d/e, the total n was divided after determination of the respective genotypes<br>- germline health (Fig. 4 g/h) was determined for n>320 individual worms over three generations and divided into the separate strains after genotyping<br>- the Mortal Germline experiment was conducted on n=15 plates per strain
Data exclusions	We excluded qFISH images when one of the pre-established criteria was met:<br>- additional telomeric signal stemming from other cell types<br>- aggregation of the probe leading to high intensity signal<br><br>We excluded worms from broodsize assays and mortal germline when one of the pre-established criteria was met:<br>- contamination of the plates leading to sickness of the worms<br>- early death of the worm due to morphological defects or crawling up the plate walls
Replication	-Results from initial MS screens were verified by DNA binding immunoprecipitation of recombinant proteins and C. elegans extract.<br>-Experiments for western blots from Fig. 1,5,6 and Supplementary Fig. 6 were performed at least twice with similar results. Exceptions are Fig. 1 c, Fig. 2a, Fig. 5b and Fig. S6h, which were not replicated. Fig. 1c depicts an expected result from a previous publication, Fig. 2a is supported by similar results from the RNAseq of Fig. S3a-c.<br>-Colocalization was checked at least twice by fluorescence microscopy, representative images of two individuals per strain from one experiment are shown in the manuscript.<br>-The qFISH experiment was repeated four independent times with different individual worms imaged, yielding similar results.<br>-The Telomere Southern Blot was repeated three independent times with genomic DNA from independent worm populations, yielding similar results.<br>-IP-qMS experiments were not repeated as the significant enrichments stem from four separate biological replicates used in the IP.<br>-The initial Y2H screen (Fig. 6a, Fig. S6) was performed twice independently with the same results. The other Y2H experiments were performed once each with elements from the other screens included as positive controls.<br>-Mortal germline and broodsize assays were performed once as n>15 of individual animals is sufficient and the positive controls used were previously published with similar generation times.<br>-Fluorescence polarization was performed at least twice for Fig. 1g/h and once for Fig.S2, yielding the overall same result.

	- Gel filtration assays were performed twice (Fig. 5a & Supplementary Fig. 6a) with the exeption of the run including treatment with Sm nuclease (Fig. 5b). This run was performed only once.
Randomization	In the establishment of the double mutants by crossing and the subsequent follow up, the worms for each generation were picked randomly from a parent with a known genotype.<br>Worms for longevity assays and mortal germline were selected randomly from a maintenance plate containing worms of the same ages and genotypes.<br>Images of germlines and embryos for qFISH analysis were taken randomly throughout the slides from randomly picked and fixed worms.
Blinding	Germline categories in the double mutant experiments were assigned without knowledge of the genotype.<br>Otherwise blinding was not relevant for this study, as for the majority of experiments the strains analyzed needed to be assigned before to be able to perform the respective experiment and was therefore not part of the experimental design.

Reporting for specific materials, systems and methods

We require information from authors about some types of materials, experimental systems and methods used in many studies. Here, indicate whether each material, system or method listed is relevant to your study. If you are not sure if a list item applies to your research, read the appropriate section before selecting a response.

Materials & experimental systems

n/a	Involved in the study	
☐	☒	Antibodies
☒	☐	Eukaryotic cell lines
☒	☐	Palaeontology and archaeology
☐	☒	Animals and other organisms
☒	☐	Human research participants
☒	☐	Clinical data
☒	☐	Dual use research of concern

Methods

n/a	Involved in the study	
☒	☐	ChIP-seq
☒	☐	Flow cytometry
☒	☐	MRI-based neuroimaging

Antibodies

Antibodies used	anti-GFP from mouse (Roche), #1181446001 (Merck), clones 7.1 & 13.1 - dilution 1:1000<br>anti-FLAG from mouse (Sigma-Aldrich), #F3165 (Merck), clone M2 - dilution 1:5000 for WB, 0.5 µg per IP sample<br>anti-Actin from rabbit (Sigma-Aldrich), #A2066 (Merck) - dilution 1:500<br>anti-His-HRP from mouse (Qiagen), part of the Penta-His HRP conjugate Kit, #34460 - dilution 1:1000<br>anti-mouse-HRP from donkey (Cell Signaling Technology), #7076 - dilution 1:10000<br>anti-rabbit-HRP (GE Healthcare), #NA934 - dilution 1:3000<br>IRDye 680RD anti-mouse IgG antibody (LI-COR), #926-68072 - dilution 1:15000<br>IRDye 800CW anti-rabbit IgG antibody (LI-COR), #926-32213 - dilution 1:15000
Validation	anti-GFP antibody quality control by Sigma-Aldrich:<br>- Western Blot: specific band from E.coli extract containing a recombinant GFP fusion protein detected at a 0.4 ug/ml antibody concentration. No non-specific binding was seen for a negative control lysate without GFP fusion protein.<br>- IP: Capture of GFP fusion protein from E. coli extract by antibody, vizualized by western blot<br>anti-FLAG: no validation statement on manufacturer's website, western blot and IP functionality were confirmed using C. elegans extract containing a FLAG fusion protein.<br>anti-actin quality control by Sigma-Aldrich: working dilutions for western blot of at least 1:100 were determined using chicken gizzard extract.<br>Anti-His HRP: no documentation on manufacturer's website, validation by western blot with E. coli lysate containing His-tagged proteins

Animals and other organisms

Policy information about studies involving animals; ARRIVE guidelines recommended for reporting animal research

Laboratory animals	Caenorhabditis elegans N2 Bristol - all mutants or tagged alleles were derived from this strain<br>-> hermaphrodite worms used for all experiments, developmental stages: embryos, larval stages L1-L4, young adults, gravid adults<br>-> male worms used for crosses<br>Caenorhabditis briggsae AF16 (hemaphrodite worms, gravid adults used for extract preparation)
Wild animals	the study did not involve wild animals
Field-collected samples	the study did not involve samples collected from the field
Ethics oversight	No ethical approval or guidance was required.

Note that full information on the approval of the study protocol must also be provided in the manuscript.
